# Blackened *Panax quinquefolius* L. Saponins and Their Cytotoxic Effect on HepG2 Cells

**DOI:** 10.3390/molecules31071173

**Published:** 2026-04-01

**Authors:** Yuanyuan Tian, Jiaqi Gao, Yongqi Liu, Rui Liu

**Affiliations:** State Key Laboratory of Food Nutrition and Safety, College of Food Science and Engineering, Tianjin University of Science and Technology, Tianjin 300457, China; yuantian899@163.com (Y.T.); gjq751421155@163.com (J.G.); l17591619923@163.com (Y.L.)

**Keywords:** *Panax quinquefolius* L., saponins, transformation, antioxidant, cell cytotoxicity

## Abstract

In the present work, the blackening process of *Panax quinquefolius* L. (PQ) was systematically investigated at temperatures of 70–90 °C, relative humidities (RHs) of 70–85%, and treatment times of 0–14 days. Ginsenoside compositions and transformation pathways were analyzed by high-performance liquid chromatography (HPLC) and liquid chromatography coupled with ion trap time-of-flight tandem mass spectrometry (LC-IT-TOF-MS/MS). The results demonstrated that blackening treatment significantly increased total saponin content from 2.72% to 5.73% after being treated at 80 °C and 70% RH for 12 days, accompanied by the highest conversion efficiencies for newly generated ginsenosides Rk1 (8.89 mg/g) and Rg5 (17.69 mg/g). Furthermore, compared with untreated PQ saponins (PQS), the blackened PQ saponins treated under optimal conditions (BPQS) exhibited superior 1,1-diphenyl-2-picrylhydrazyl (DPPH) and 2,2′-azino-bis(3-ethylbenzothiazoline-6-sulfonic acid) cation (ABTS^+^) radical scavenging activities, with IC_50_ values of 0.2999 mg/mL and 0.2640 mg/mL, respectively, as well as stronger reducing power. Meanwhile, BPQS exhibited higher cytotoxicity toward HepG2 cells and effectively inhibited cell survival and proliferation by promoting the expression of apoptosis-related proteins, including caspase 3 and caspase 9. Our findings indicate that BPQS may be a functional ingredient suitable for use in dietary supplements and disease chemoprevention.

## 1. Introduction

*Panax quinquefolius* L. (PQ), also known as American ginseng, is a perennial herbaceous plant in the ginseng genus of the Araliaceae family, native to North America and from areas such as Montreal and Vancouver in Canada [1,2]. It has been subsequently cultivated in more than ten provinces of China since the late 18th century. Pharmacological studies have indicated that PQ possesses various biological activities, such as regulating glucose metabolism, protecting the cardiovascular system, modulating the endocrine system, and exerting antioxidant, anti-diabetic, anti-inflammatory, anti-tumor, anti-aging, anti-fatigue, and neuroprotective effects [3,4,5,6]. The benefits of these metabolic and physiological responses come from a variety of bioactive compounds in PQ, including saponins, polysaccharides, amino acids, sterols, and proteins [7,8].

Triterpene saponins, called ginsenosides or ginseng saponins, are the predominant bioactive phytochemicals of PQ, consisting of aglycone moiety structures linked to one or more oligosaccharide or glucuronic acid units [6,9]. According to their aglycone structures, ginsenosides can be classified into dammarane-, oleanane-, and ocotillol-type saponins [10]. Dammarane-type saponins include ginsenosides Rb1, Rb2, Re, and Rg1, which account for a majority of saponins in PQ [11]. They and their derivatives can be mainly divided into protopanaxadiol (PPD)-type (Rb1, Rb2, Rd, F2, Rg3, Rk1, Rg5, Rh2, etc.) and protopanaxatriol (PPT)-type (Re, Rg1, Rg2, F1, F3, Rh1, etc.), based on the structure differentiations in the number and position of hydroxyl groups [9,12,13].

Although PQ primary ginsenosides such as Re, Rb1, Rg1, and Rd accounted for the majority, rare ginsenosides including Rh2, Rg3, Rg5, and Rk1 generally exhibited superior bioactivity, less polarity, and are more easily absorbed by the human body [14,15,16]. Therefore, the degradation of ginsenosides has been achieved through extensively used physicochemical and biological strategies, including heating, acid hydrolysis, microwave irradiation, and enzymatic and microbial transformations [14,17,18]. Among them, heating and acid hydrolysis are widely used for the transformation of ginsenosides, owing to their low cost and high yield in the production of rare ginsenosides with diverse structures [9]. A prime example of thermal processing is “black ginseng”, which is produced by repeated steaming and drying. This process enhances its medicinal properties compared to white or red ginseng, because the ginsenosides undergo reactions such as hydrolysis, dehydration, decarboxylation, and isomerization, transforming into rare ginsenosides [19,20]. In fact, the “black” in black ginseng is the result of the Maillard reaction, a chemical process between reducing sugars and amino acids that produces glycosylamines and/or ketosamines [21], which are significantly influenced by the specific conditions, such as temperature, water activity (relative humidity, RH), and reaction time (or process residence time) [22]. Therefore, the blackening of PQ should be precisely regulated at different temperatures and RHs. A rapid transformation method has been developed to prepare rare dehydroxylated ginsenosides by heating and acid treatment (120 °C, 4 h, 0.01% formic acid); from 100 mg of Rg3, Rk1 (7.4 mg), and Rg5 (15.1 mg) can be produced, and Rk1 exerted dominant chemotherapeutic activities [23]. Inspired by this, similar heating and acid treatments have been directly applied to PQ material itself, and results showed that this treatment promoted the transformation of polar ginsenosides to less-polar ginsenosides in PQ; the total ginsenoside content was approximately 10.98 mg/g, with the main ginsenosides including Rh3 (2.72 mg/g), Rk2 (2.21 mg/g), Rk1 (1.38 mg/g), Rb1 (1.22 mg/g), and (R)-Rg3 (1.06 mg/g) [24]. By using aspartic acid as a catalyst and thermal extraction, the highest conversion rates of Rk1 (6.58 mg/g) and Rg5 (3.74 mg/g) were achieved in PQ, and ginsenosides in combination with CTX could significantly upregulate the expressions of Bax and cleaved-caspase 3 and inhibit the expression of anti-apoptotic protein Bcl-2 [25]. However, during the blackening process of *Panax quinquefolius* L., there are still challenges such as the unclear transformation pathways and the lack of clear biological activity and efficacy.

Liquid chromatography coupled with ion trap time-of-flight tandem mass spectrometry (LC-IT-TOF-MS/MS) integrated the advantages of TOF-MS and IT-MS, producing sophisticated and high-accuracy ion fragments, thereby enabling efficient qualitative inference of disintegration patterns [26]. For cytotoxicity and biological assays, the Cell Counting Kit-8 (CCK-8) assay and flow cytometry using Annexin V-FITC/PI dual staining has been extensively applied in the measurement of cell viability and apoptosis [27,28]. Caspase 9 and caspase 3 are the initiator and effector caspases of the intrinsic apoptosis pathway, respectively. Detecting the activities of caspase 3 and caspase 9 will provide direct evidence for cell apoptosis [29].

Our previous research showed that both blackening color difference value and total ginsenosides content in *Panax ginseng* reached their maximum values at 80 °C and 75% RH after 10 days of treatment [30]. However, in PQ, the blackening rate was much faster than that of rare ginsenosides transformation, indicating that the blackening process was mainly ascribed to raw material compositions (e.g., total sugar, reducing sugar, and protein content) and processing conditions (e.g., temperature and RH). Therefore, we systematically studied the blackening process of *Panax quinquefolius* L. under different temperatures, relative humidities, and treatment times. The potential transformation pathways of PQ ginsenosides were analyzed using liquid chromatography coupled with ion trap time-of-flight tandem mass spectrometry (LC-IT-TOF-MS/MS). The antioxidant activities were evaluated by measuring the scavenging rates of 1,1-diphenyl-2-picrylhydrazyl (DPPH) and 2,2′-azino-bis(3-ethylbenzothiazoline-6-sulphonic acid) cation (ABTS^+^), as well as the total reducing power. Meanwhile, the cytotoxic effect on HepG2 cells was investigated by assessing cell viability, apoptosis, and the expression of apoptosis-related proteins.

## 2. Results and Discussion

### 2.1. Blackening Treatment Improved Total Saponin Content in Panax quinquefolius L.

*Panax quinquefolius* L. was processed via blackening at different temperatures (70–90 °C) and relative humidities (RHs, 70–85%) for 0–14 days. As shown in Table 1, except at 70 °C, the total saponin content of black ginseng samples at 80 °C and 90 °C generally first increased and then decreased with the extension of treatment time (0–14 days), regardless of relative humidity (RH). At 70 °C, however, the total saponin content showed a decreasing trend. From day 2 onward, the total saponin content of black ginseng samples at 90 °C was higher than that of untreated ginseng; from day 4 onward, the total saponin content at 80 °C generally exceeded that of untreated ginseng. This result was consistent with the report by Jang et al. [31], which stated that moderately high temperatures (110 and 130 °C) typically increased the ginsenoside content, but an excessive heat treatment at 150 °C could cause the loss of ginsenoside content owing to the thermal degradation effect. Specifically, compared with the untreated sample, black ginseng samples under the following conditions showed significantly higher total saponin content (>5%): 80 °C, 70% RH for 6 d (5.21% ± 0.85%); 80 °C, 85% RH for 6 d (5.29% ± 0.07%); 90 °C, 70% RH for 6 d (5.09% ± 0.43%); 80 °C, 85% RH for 8 d (5.29% ± 0.32%); 80 °C, 70% RH for 12 d (5.73% ± 0.15%); 90 °C, 75% RH for 14 d (5.08% ± 0.25%).

It was noteworthy that the polysaccharide content of black ginseng samples generally first increased and then decreased as the treatment time increased (0–14 days), regardless of temperature and relative humidity (RH). As shown in Table S2, at 70 °C and 80 °C, the changes in polysaccharide content showed no obvious pattern with RH; however, at 90 °C, the polysaccharide content generally first increased and then decreased with rising RH. Compared with the untreated sample, black ginseng samples had relatively higher polysaccharide content (>13%): 80 °C, 80% RH for 8 d (13.14% ± 0.73%); 80 °C, 70% RH for 10 d (14.34% ± 0.14%); 80 °C, 80% RH for 10 d (13.20% ± 0.87%); 80 °C, 70% RH for 12 d (13.06% ± 0.51%); 70 °C, 70% RH for 14 d (14.32% ± 0.54%). Nevertheless, a significantly low water content (<10%) was usually obtained at 90 °C and 70% RH (Table S3).

To sum up, blackening treatment significantly (*p* < 0.05) improved the total saponin and polysaccharide contents of ginseng samples. The total saponin content of black ginseng reached its maximum value of 5.73% ± 0.15% after being treated at 80 °C and 70% RH for 12 days, accompanied by a relatively high polysaccharide content of 13.06% ± 0.51%. Saponin extracts obtained from untreated *Panax quinquefolius* L. were assigned as PQS, while saponins extracted from blackened *Panax quinquefolius* L. under optimal conditions were named BPQS.

### 2.2. Blackening Treatment Altered the Ginsenoside Compositions of Panax quinquefolius L.

**Figure 1:**
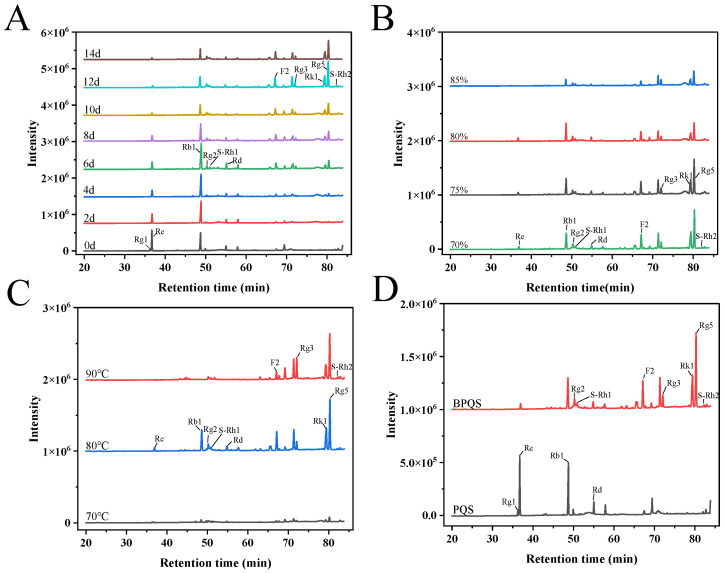
Ginsenoside changes in blackened *Panax quinquefolius* L. treated at different temperatures (70–90 °C) and relative humidities (RHs, 70–85%) for 0–14 days. HPLC elution curves of ginsenosides in black ginseng treated at 80 °C and 70% RH for 0–14 days (**A**), 80 °C and 70–85% RH for 12 days (**B**), and 70–90 °C and 70% RH for 12 days (**C**); HPLC elution curves of ginsenosides in untread *Panax quinquefolius* L. (PQS) and blackened *Panax quinquefolius* L. (BPQS) treated at 80 °C and 70% RH for 12 days (**D**).

The alterations in 11 ginsenosides of *Panax quinquefolius* L. during the blackening process were quantitatively analyzed by HPLC. As shown in Figure 1A, the contents of ginsenosides Re and Rg1 gradually decreased as the treatment time increased, while Rb1 content showed a rising trend first and then falling over time. The content of ginsenoside Rb1 reached its maximum value of 18.93 mg/g after 6 days of processing, but gradually declined to 8.40 mg/g after 12 days of treatment. The initial increase in Rb1 content up to day 6 might be attributed to the enhanced release and extractability of ginsenosides under appropriate temperature and humidity [30]. The subsequent decrease after day 6 was consistent with the kinetic behavior of ginsenoside Rb1 thermal degradation. Kwon et al. [32] established a kinetic model for Rb1 thermal degradation, demonstrating that Rb1 underwent consecutive conversion reactions with a kinetic rate constant of 0.013 h^−1^ at 80 °C, first to Rg3 and then further to dehydrated products including Rk1 and Rg5. Yao et al. [33] similarly reported that the content of polar ginsenosides, including Rb1, decreased progressively with prolonged thermal treatment. Compared with untreated ginseng, the content of ginsenosides Rk1 and Rg5 in black ginseng treated at 80 °C and 70% RH for 12 days increased significantly, reaching 8.89 mg/g and 17.69 mg/g, respectively. Figure 1B shows the changes in black ginseng saponins, varying the RHs of 70–85% at 80 °C for 12 days. The result indicated that the content of rare ginsenosides such as Rk1 and Rg5 was higher at RHs of 70% and 75% compared to 80% and 85%. Figure 1C shows the changes in black ginseng saponins with varying temperatures of 70–90 °C at 70% RH for 12 days. Higher temperature resulted in relatively high conversion efficiency, with Re and Rg1 being completely converted at 90 °C. At 90 °C, the content of ginsenoside Rg3 was higher than at 70 °C and 80 °C. However, Rk1 and Rg5 were enriched at the moderate temperature of 80 °C. Finally, the ginsenoside compositions of PQS and BPQS were comparatively analyzed, as shown in Figure 1D. As shown in Table S4, compared with PQS, ginsenoside Rg1 in BPQS was completely transformed. The content of polar ginsenosides Re, Rb1, and Rd decreased from 11.18 mg/g, 10.37 mg/g, and 3.20 mg/g to 1.25 mg/g, 8.40 mg/g, and 1.75 mg/g, respectively, while less-polar ginsenosides F2, Rg3, Rk1 and Rg5 increased significantly, reaching 7.92 mg/g, 1.77 mg/g, 8.89 mg/g, and 17.69 mg/g, respectively. The total amount of 11 ginsenosides also increased significantly, rising from 25.91 mg/g to 50.52 mg/g. These results indicated that the chemical bonds in ginsenosides were broken during heat treatment, converting them into less polar ginsenosides [19]. Additionally, blackening parameters, including temperature, relative humidity, and time, might also influence the transformation pathways of ginsenoside compositions [34]. For instance, Park et al. [35] previously processed *Panax quinquefolius* L. by steaming ginseng extract at 120 °C and 0.11 MPa for 3 h, followed by drying at 50 °C for 3 days. The resulting ginseng saponins mainly contained ginsenosides (S)-Rg3 (11.3 mg/g), (R)-Rg3 (3.9 mg/g), Rk1 (2.9 mg/g), and Rg5 (3.6 mg/g), which differed from the ginsenoside composition in this study. In addition, Li et al. [25] treated *Panax quinquefolium* L. using four different methods, including reflux, heating, soaking, and ultrasonic extraction; the results showed that heating extraction under optimal conditions achieved the highest conversion of Rk1 (6.58 mg/g) and Rg5 (3.74 mg/g).

### 2.3. Potential Transformation Pathways of Ginsenosides During Blackening Treatment

To speculate on the transformation pathways of ginsenosides during blackening, HPLC-IT-TOF-MS/MS was used to identify 11 ginsenosides in PQS and BPQS, combined with the HPLC analysis result and the literature [34,36]. As shown in Table 2 and Table S4, ginsenosides Rg1, Re, Rb1, and Rd were identified in PQS, and they gradually converted to less-polar ginsenosides identified in BPQS, such as (S)-Rh1, F2, Rg3, Rk1, Rg5, and (S)-Rh2. Among them, ginsenosides (S)-Rh1, Rg3, Rk1, Rg5, and (S)-Rh2 were only detected in BPQS but not in PQS, with Rk1 and Rg5 having the highest content after blackening, implying that Rk1 and Rg5 had good thermal stability, and might be the final products of ginsenosides transformation during blackening [33].

**Figure 2:**
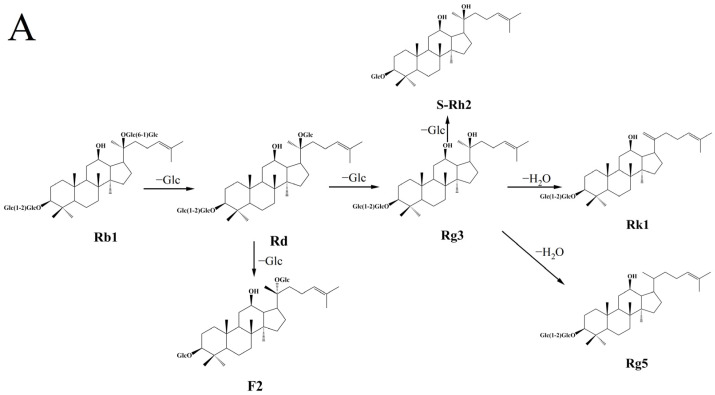

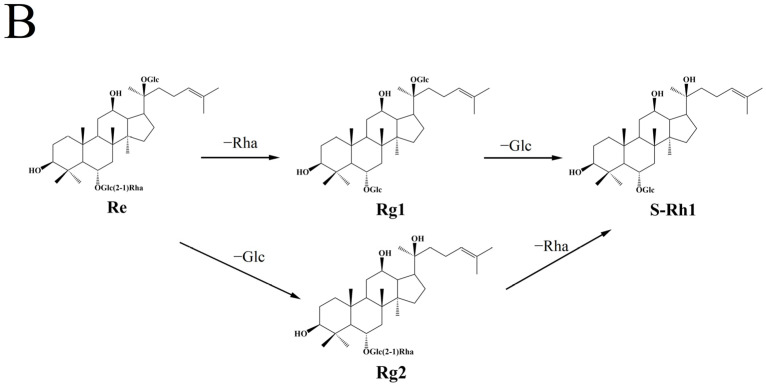
The transformation pathways of protopanaxadiol (PPD)-type ginsenosides (**A**) and protopanaxatriol (PPT)-type ginsenosides (**B**).

The transformation pathways of 11 ginsenosides during blackening are given in Figure 2. The 11 ginsenosides were classified into protopanaxadiol (PPD)-type ginsenosides (Rb1, Rd, F2, (S)-Rh2, Rg3, Rk1, and Rg5) and protopanaxatriol (PPT)-type ginsenosides (Re, Rg1, Rg2, and (S)-Rh1). The contents of seven PPD-type ginsenosides ranked as Rb1 > Rd > F2 > [(S)-Rh2, Rg3, Rk1, and Rg5] in PQS, while the contents of four PPT-type ginsenosides ranked in the order as Re > Rg1 > [Rg2 and (S)-Rh1] in untreated ginseng (Table S4). Table 2 and Figure S1 show the MS/MS identification results of ginsenoside extracts in negative ion mode, taking PPD-type ginsenosides Rb1 (Figure S1A) and Rd (Figure S1B), and PPT-type ginsenosides Re (Figure S1C) and Rg1 (Figure S1D) as examples. Ginsenoside Rb1 had a retention time (Rt) of 46.047 min and a quasi-molecular ion *m*/*z* of 1107.5927 [M−H]^−^, possessing a glycoside moiety linked with four glucose units (−Glc, 162.05 Da). The MS/MS spectrum generated four ion peaks at *m*/*z* 945.5381 [M−H−Glc]^−^, 783.4885 [M−H−2Glc]^−^, 621.4341 [M−H−3Glc]^−^, and 459.3843 [M−H−4Glc]^−^, where the four glucose residues were consecutively eliminated from the quasi-molecular ion, generating a PPD-type final product ion at 459.3843 [PPD−H]^−^. The retention time of ginsenoside Rd was 52.245 min, with an *m*/*z* of 945.5386 [M−H]^−^, where its quasi-molecular ion was probably formed by Rb1 losing a glucose unit at the C-20 position. Based on previous studies [25,37], we speculate that PPD-type ginsenoside Rb1 is converted to Rd by cleaving a sugar moiety at C-20, and Rd loses a sugar moiety at C-20 and C-3 to form Rg3 and its isomer F2, respectively; Rg3 further undergoes dehydration at C-20 to produce the isomers Rk1 and Rg5 (Figure 2A). Similarly, ginsenoside Re exhibited 34.198 min Rt, with its quasi-molecular ion *m*/*z* of 945.5356 [M−H]^−^. The glycoside moieties of Re involved three glucose units and one rhamnose unit, resulting in four ion peaks at *m*/*z* 799.4837 [M−H−Rha]^−^, 783.4887 [M−H−Glc]^−^, 637.4312 [M−H−Glc−Rha]^−^, and 475.3818 [M−H−2Glc−Rha]^−^. The result indicated that its quasi-molecular ion lost the rhamnose unit (−Rha, 146.06 Da) and then successively cleaved two glucose units, generating the PPT-type ion characteristic peak at *m*/*z* 475.3818 [PPT−H]^−^. The retention time peak of ginsenoside Rg1 appeared at 34.053 min, and its quasi-molecular ion *m*/*z* was 799.4854 [M−H]^−^, which might originate from the cleavage of the rhamnose unit in ginsenoside Re. According to [34], we hypothesize that PPT-type ginsenoside Re is converted into Rg1 or Rg2 by the loss of a rhamnose residue at C-6 or a glucose residue at C-20, respectively; Rg1 or Rg2 further undergoes hydrolysis of glucose or rhamnose residues to produce (S)-Rh1 (Figure 2B). It is important to highlight that the transformation pathways observed in this study for *Panax quinquefolius* differ from those previously reported for *Panax ginseng* under similar heat-moisture treatment [30]. While *Panax ginseng* predominantly underwent hydrolysis to produce Rg3 and CK, *Panax quinquefolius* exhibited a greater propensity for dehydration at the C-20 position, leading to the formation of Rk1 and Rg5. This discrepancy may be attributed to the inherent differences in the initial ginsenoside composition between the two species and their distinct responses to thermal processing.

Above all, during the blackening process, PPD-type ginsenosides mainly convert Rb1 into rare ginsenosides containing Rk1 and Rg5 through hydrolysis and dehydration, while PPT-type ginsenosides are easily hydrolyzed under heating conditions, converting Re into (S)-Rh1 [38]. The transformation pathway results indicated that blackening of *Panax quinquefolius* L. mainly involved deacetylation, deglycosylation, and dehydration of polar ginsenosides to form less-polar ginsenosides, which may facilitate the release of bioactive components with good absorption, thereby enhancing its nutritional and biological functions.

### 2.4. Blackening Treatment Improved In Vitro Antioxidant Activities of Panax quinquefolius Saponins

**Figure 3:**
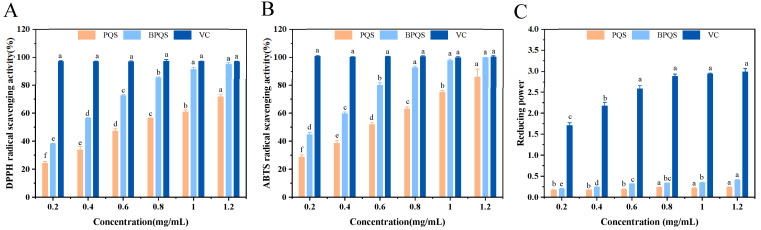
DPPH radical scavenging ability (**A**), ABTS radical scavenging ability (**B**), and total reducing power (**C**) of different concentrations (0.2–1.2 mg/mL) of PQS and BPQS. L-Ascorbic acid (VC) was used as a positive control. Different lowercase letters (a–f) indicated a significant difference among different concentrations (*p* < 0.05).

The scavenging activities of DPPH and ABTS^+^ radicals, and total reducing power were used to evaluate the antioxidant activities of PQS and BPQS. As shown in Figure 3, the antioxidant activities of PQS and BPQS both displayed an obvious dose-dependent manner. DPPH radical scavenging assay showed that the half-maximal inhibitory concentrations (IC_50_ values) of PQS and BPQS were 0.6372 mg/mL and 0.2999 mg/mL, respectively; at the same concentration, BPQS exhibited significantly (*p* < 0.05) higher DPPH radical scavenging ability than that of PQS (Figure 3A). Furthermore, Figure 3B showed that the IC_50_ values of PQS and BPQS for ABTS^+^ radical scavenging were 0.4964 mg/mL and 0.2640 mg/mL, respectively, indicating that BPQS possessed stronger ABTS^+^ scavenging ability, compared with PQS. Figure 3C indicated that both PQS and BPQS had considerable reducing ability, despite a very high reducing power of L-Ascorbic Acid (VC). The result was inconsistent with previous studies of [30,39,40,41], which suggested that *Panax quinquefolius* saponins exhibited good antioxidant activity, and might be an effective strategy for ameliorating oxidative stress related with a wide range of diseases including neurodegenerative disorders, diabetes, cancer, etc. For example, Li et al. [42] evaluated the differences in quality of low-temperature softened (LTS-HAD), blanched (BL-HAD), steaming and hot-air drying (ST-HAD), and vacuum freeze-dried (VFD) ginseng, which indicated that ST-HAD samples displayed reddish-brown color and the highest antioxidant activity, with DPPH, ABTS^+^, and FRAP IC_50_ values of 5.55 mg/mL, 1.41 mmol TROLOX/g, and 0.41 mM Fe(II)/g, respectively. Interestingly, the highest antioxidant activity was achieved and probably accompanied by the presence of (S)-Rg2, (R)-Rg2, Rg6, (S)-Rg3, (R)-Rg3, Rk1, and Rg5. The radical scavenging and reducing ability of ginsenosides might be attributed to the structural changes occurring at the C-20 position [43]. Also, Choi et al. [44] applied microwave irradiation to enhance the anticancer activity of ginseng, while the levels of Rg3, Rg5, and Rk1 increased via structural conversion of ginsenosides. 

### 2.5. Blackening Treatment Enhanced Cell Cytotoxicity of Panax quinquefolius Saponins on HepG2 Cells

#### 2.5.1. Cell Cytotoxicity

**Figure 4:**
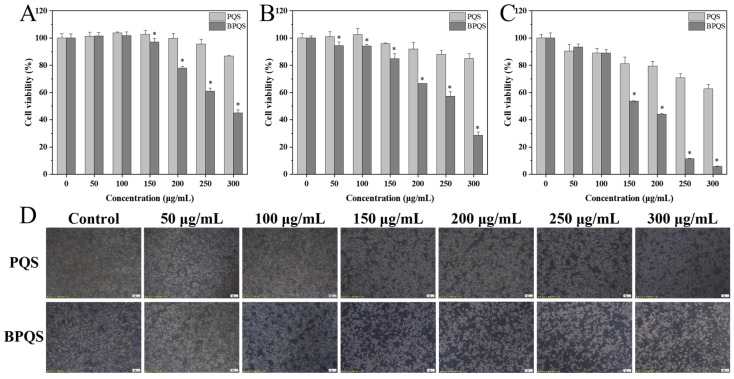
Cell viability of different concentrations (0–300 μg/mL) of PQS and BPQS against HepG2 cells after incubation for 12 h (**A**), 24 h (**B**), and 48 h (**C**), respectively; the growth state (**D**) of HepG2 cells treated with different concentrations (0–300 μg/mL) of BQS and BPQS. * indicated a significant difference between PQS and BPQS (*p* < 0.05).

The Cell Counting Kit-8 (CCK-8) assay has been extensively applied in the measurement of cell viability and cytotoxicity [27]. In this study, we applied the CCK-8 assay to evaluate the effect of blackening on the cell cytotoxicity of *Panax quinquefolius* saponins by assessing the cell viability of PQS and BPQS at various concentrations (0–300 μg/mL) against HepG2 cells. Figure 4A shows that after 12 h of incubation, there was no significant difference in the effects of PQS and BPQS on HepG2 cell viability at concentrations below 150 μg/mL. However, when the concentration exceeded 150 μg/mL, the cell viability of BPQS was significantly lower than that of PQS. After 24 h of incubation, the IC_50_ values of PQS and BPQS were 586.6 μg/mL and 249.6 μg/mL, respectively (Figure 4B); after 48 h of incubation, the IC_50_ value of BPQS (168.0 μg/mL) was significantly lower than that of PQS (545.3 μg/mL), indicating that BPQS could achieve half-maximal inhibition at a lower dose compared to PQS (Figure 4C). Thus, 48 h of incubation was selected for subsequent experiments. The results suggested that, compared with PQS, BPQS exhibited a stronger inhibitory effect on HepG2 cells, having a time- and dose-dependent behavior. Based on previous results and the literature [44,45], it can be concluded that blackening significantly enhances the inhibitory effect of BPQS on cell viability, which may be related to increased total saponin content and levels of less-polar ginsenosides Rg5 and Rk1.

#### 2.5.2. Cell Growth State

The effect of PQS and BPQS (0–300 μg/mL) on the growth state of HepG2 cells incubated for 48 h was observed using an inverted microscope (10×). As shown in Figure 4D, the HepG2 cells in the control group had smooth surfaces, regular shapes, tight intercellular connections, healthy growth, and firm adherence, indicating that cells could proliferate normally in the absence of PQS and BPQS. Compared with the control group, HepG2 cells treated with PQS and BPQS both showed obvious dose-dependent growth inhibition. At low concentrations of 0–100 μg/mL, there was no significant difference in the cell morphology of HepG2 cells treated with PQS and BPQS. However, as the concentration of saponin extract increased (150–300 μg/mL), the cell edges gradually became blurred, and intercellular connections loosened; the BPQS-treated group showed a stronger inhibitory effect on the growth of HepG2 cells, manifested by severe shrinkage, reduced cell volume, decreased adhesion and floating in the culture medium, with some cells even breaking into granular forms (250–300 μg/mL). This indicated severe apoptosis or necrosis, consistent with the results of the cell cytotoxicity assay. Additionally, the results of cell migration (Figure S2 and Table S5) illustrated that when the concentrations of PQS and BPQS reached 250 μg/mL and 150 μg/mL, respectively, the saponin extract administration caused cell shrinkage and volume reduction, leading to a scratch area larger than the initial scratch area, thereby resulting in a negative cell migration rate. In particular, at BPQS concentrations of 250 μg/mL and above, severe cell shrinkage and floating phenomena were observed, and the scratch area was not apparent. Based on the observations of cell growth state and cell migration results, concentrations of PQS and BPQS in the range of 0–200 μg/mL were chosen for incubation of HepG2 cells for 48 h to further investigate cell apoptosis and Western blot analysis.

#### 2.5.3. Cell Apoptosis

**Figure 5:**
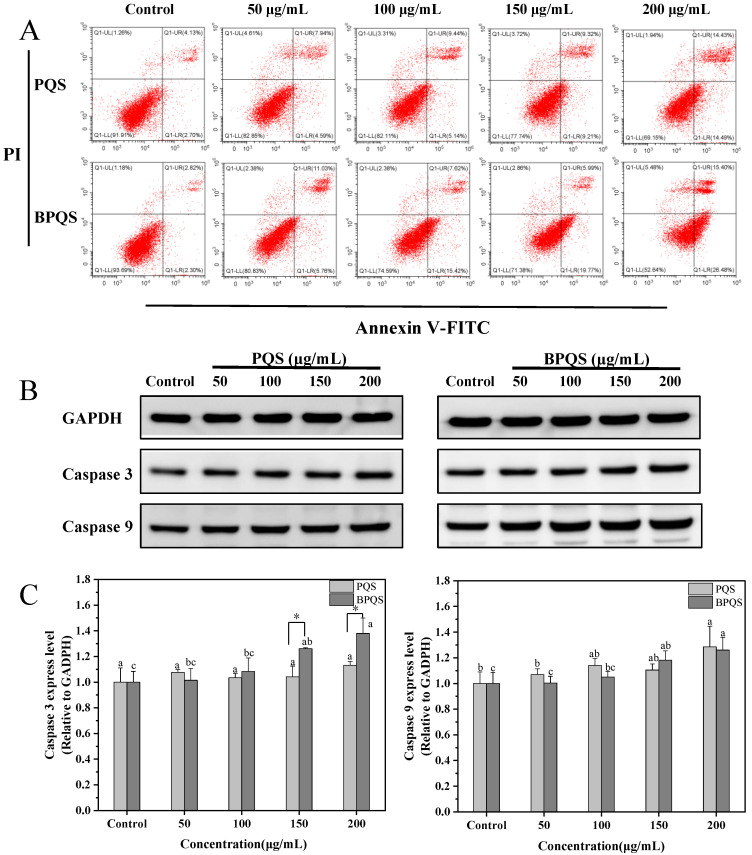
Cell apoptosis rate (**A**) of different concentrations (0–200 μg/mL) of PQS and BPQS against HepG2 cells after incubation for 48 h. Western blot images (**B**) and the expression levels (**C**) of apoptosis-related proteins caspase 3 and caspase 9 in HepG2 cells treated with different concentrations (0–200 μg/mL) of PQS and BPQS after incubation for 48 h. Different lowercase letters (a–c) indicated a significant difference among different concentrations (*p* < 0.05). * indicated a significant difference between PQS and BPQS (*p* < 0.05).

The apoptosis rate of HepG2 cells treated with different concentrations (0–200 μg/mL) of PQS and BPQS was detected using Annexin V-FITC/PI dual staining by flow cytometry (Figure 5A). Compared with the control group, both PQS and BPQS efficiently induced cell apoptosis in a dose-dependent manner. Within the range of 0–200 μg/mL saponin extract concentration, the early apoptotic cell rate in the PQS group and BPQS group increased from approximately 2.70% and 2.30% to 14.49% and 26.48%, respectively; their late apoptotic cell rate rose from 4.13% and 2.82% to 14.43% and 15.40%, respectively. The results showed that at the concentration of 200 μg/mL, BPQS effectively inhibited the survival and proliferation of HepG2 cells with a cell apoptosis rate of 41.88%, significantly higher than PQS′s 28.92%. Considering the transformation of saponins during blackening, the better apoptosis-inducing effect of BPQS might be attributed to its relatively high content of less-polar ginsenosides such as Rk1 and Rg5. Previous studies have indicated that rare ginsenosides Rg5 and Rk1 can promote tumor cell apoptosis by regulating apoptosis-related signaling pathways such as mitochondrial pathways and the caspase cascades [25,30,45]. Therefore, the presence of rare ginsenosides Rk1 and Rg5 in BPQS may be one of the key factors contributing to its enhanced antitumor activity in HepG2 cells. It should be noted that the safety of BPQS could be further evaluated using a normal cell line, for example, LO2 or THLE-2, etc. [46,47].

#### 2.5.4. Western Blot Analysis

Caspases are a family of cysteine proteases that participate in the cascade reactions involved in processes such as cell apoptosis, inflammation, necroptosis, and pyroptosis [48,49]. Caspase 9 is an initiator caspase of the intrinsic apoptosis pathway, and its activation further triggers the activation of the effector caspase 3 [29]. Activated caspase 3, in turn, cleaving specific downstream cellular substrates leads to the disintegration of cellular structures, ultimately causing cell death [50,51]. To understand the apoptosis regulation of PQS and BPQS, the expression levels of apoptosis-related proteins caspase 3 and caspase 9 in HepG2 cells were determined by Western blot (Figure 5B). The results indicated that both PQS and BPQS upregulated the expression level of caspase 9 in a dose-dependent manner; however, at the same concentration, there was no significant difference in caspase 9 expression levels between PQS and BPQS (*p* > 0.05). Besides, as the concentration increased (0–200 μg/mL), BPQS also upregulated the expression levels of caspase 3. It is noteworthy that PQS had no significant effect on caspase 3 expression. (Figure 5C) This might be because in this model: 1. compensatory transcriptional mechanisms are only activated when the apoptotic signal exceeds a certain threshold, thereby increasing the total caspase protein level [52]; 2. PQS-induced apoptosis may also involve other executive proteases, such as caspase-7 [53]. When the concentration reached 150 μg/mL or above, the expression level of caspase 3 in HepG2 cells treated with BPQS was significantly higher than that in the PQS-treated group (*p* < 0.05), indicating that BPQS induced cell apoptosis through the caspase 3 and caspase 9-related intrinsic apoptotic pathway in HepG2 cells [54].

## 3. Materials and Methods

### 3.1. Materials

Six-year-old fresh *Panax quinquefolius* L. was purchased from the local market in Tonghua (Jilin Province, China), and the chemical compositions of *Panax quinquefolius* L. were provided in Table S1. Ginsenoside reference standards, including Re, Rg1, Rg2, Rg3, (S)-Rh1, (S)-Rh2, Rb1, Rd, F2, Rk1, and Rg5 (HPLC purity ≥ 98%), were purchased from Chengdu Must Bio-Technology Co., Ltd. (Chengdu City, China). The cell counting kit-8 (CCK-8) and Annexin V/PI dual staining reagent were purchased from Beyotime Biotech Inc. (Shanghai, China). Mouse Caspase 3, Caspase 9, and GAPDH assay kits, and HRP-conjugated goat anti-mouse IgG were purchased from Proteintech Group (Chicago, IL, USA). Unless otherwise noted, all other chemicals and reagents were used at HPLC-grade or analytical-grade as required.

### 3.2. Blackening Process of Panax quinquefolius L.

The blackening process of fresh ginseng was performed according to the method of Liu et al. [30] with slight modifications. Briefly, fresh ginseng was washed and drained of excess surface water, followed by a blackening process at different treatment temperatures (70, 80, and 90 °C) and relative humidities (RHs, 70%, 75%, 80%, and 85%) for 14 days. The blackened ginseng was dried at 60 °C and ground to pass through a 60-mesh sieve for subsequent experiments.

### 3.3. Determination of Total Saponin Content

The content of total saponins in untreated *Panax quinquefolius* L. and blackened *Panax quinquefolius* L. was determined according to the method of Liu et al. [30] with some modifications. In short, the ginseng sample (1 g) was mixed with 80% methanol aqueous solution (methanol:water = 80:20, *v*/*v*) at a solid-to-liquid ratio of 1:20, then subjected to ultrasonic treatment (30 min) three times. All the extracts were combined and concentrated to dryness using a RE-52 rotary evaporator (Yarong Biochemical Instrument Co., Ltd., Shanghai, China). The residue was dissolved in 20 mL of distilled water, extracted with 10 mL of water-saturated n-butanol in a separatory funnel, followed by shaking thoroughly for 30 min and allowing it to stand for another 30 min. The extraction process was repeated three times. Then, the upper n-butanol layers were collected, washed twice with 20 mL of distilled water, concentrated to dryness, and made up to 10 mL with methanol to obtain the total saponin extract. Specifically, the total saponin extracts of untreated *Panax quinquefolius* L. and blackened *Panax quinquefolius* L. treated at 80 °C and 70% RH for 12 days were referred to as PQS and BPQS, respectively. The total saponin content was determined by using the vanillin-sulfuric acid colorimetric method [55]. In brief, the standard ginsenoside Re solution (1 mg/mL) was prepared using methanol as the standard solvent. A series of Re standard solutions (0, 10, 20, 40, 60, 80, and 100 μL) was absorbed into a plug test tube, steamed to dryness in a water bath at 60 °C, and then added with 0.5 mL of 8% vanillin and 5 mL of 72% sulfuric acid. Then, the mixed solutions were thoroughly vortexed and kept in a 60 °C water bath for 10 min, followed by cooling in an ice-water bath for 10 min. The absorbance at 544 nm was measured to plot the standard curve (y = 5.07397x − 0.00182, R^2^ = 0.99222).

### 3.4. High-Performance Liquid Chromatography (HPLC) Analysis

The ginsenoside profiles of untreated and blackened ginseng were analyzed by using an LC-20A HPLC system (Shimadzu, Kyoto, Japan) equipped with an SPD-20A UV detector, an Agilent 5HC-C18 column (250 × 4.6 mm, 5 μm), a LC-20AD binary pump, an SIL-20A autosampler, a CTO-20A column oven, and an DGU-20A online degassing unit. Following in the method of Chen et al. [56] with slight modifications, the gradient elution program was set as 18.5% B at 0 min, 20.5% B at 20 min, 25.5% B at 30 min, 35% B at 40 min, 45% B at 60 min, 60% B at 70 min, 70% B at 80 min, 80% B at 90 min, 18.5% B at 91 min, and 18.5% B at 95 min. The mobile phase comprised ultrapure water as the A phase and acetonitrile as the B phase at a flow rate of 1.0 mL/min. The column temperature was 30 °C, and the injection volume was 20 μL. The determination wavelength was set as 203 nm.

### 3.5. Liquid Chromatography Coupled with Ion Trap Time-of-Flight Tandem Mass Spectrometry (LC-IT-TOF-MS/MS) Analysis

Ginsenosides and their transformation pathways during ginseng blackening were identified and analyzed by using a Shimadzu LCMS-IT-TOF tandem mass spectrometry (MS) system. HPLC analysis employed the conditions as described in Section 3.4. MS/MS parameters were set as follows: precursor and secondary *m*/*z*, 150–1200 Da; heat block temperature and the curved desolvation line (CDL) temperature, 200 °C; electrospray ionization (ESI) source in a negative ion mode; nebulizing gas (N_2_) flow rate, 1.5 L/min; drying gas pressure, 110 kPa; detector voltage, 1.57 kV; injection volume, 3 μL; full scan mode. Following the methods of Liu et al. [30] and Liu et al. [57], the ion fragment signals were recorded and automatically chosen by Shimadzu LCMSsolution Ver3 software based on their intensity.

### 3.6. Determination of In Vitro Antioxidant Activities

#### 3.6.1. DPPH Radical Scavenging Ability

The DPPH radical scavenging ability (RSA) of PQS and BPQS was determined according to the method reported by Ning et al. [58], with slight modifications. In brief, 100 μL of PQS and BPQS ethanol solutions at different concentrations (0.2, 0.4, 0.6, 0.8, 1.0, and 1.2 mg/mL) were well mixed with 100 μL of 0.1 mM DPPH methanol solution in a 96-well plate and reacted in the dark at room temperature for 30 min. The absorbance of the PQS and BPQS samples was recorded at 517 nm (*A*_1_), and L-Ascorbic Acid (VC) was used as a positive control. Anhydrous ethanol instead of the PQS and BPQS sample was recorded as *A*_0_, while anhydrous ethanol instead of the DPPH solution was recorded as *A*_2_. The DPPH RSA was calculated by the following equation:

DPPH RSA (%) = [1 − (*A*_1_ − *A*_2_)/*A*_0_] × 100

#### 3.6.2. ABTS^+^ Radical Scavenging Ability

ABTS^+^ radical scavenging ability (RSA) of PQS and BPQS was measured following the method of Ren et al. [59] with some modifications. In brief, ABTS (7 mM) was reacted with potassium persulfate (2.45 mM) at a ratio of 1:1 in darkness at room temperature for 14 h. The ABTS working solution was prepared by diluting the reacting solution with anhydrous ethanol until the absorbance at 735 nm reached 0.700 ± 0.02. Then, 50 μL of the PQS and BPQS sample solution at various concentrations (0.2–1.2 mg/mL) was mixed with 200 μL of the ABTS working solution and reacted in the dark for 10 min. The absorbance of the PQS and BPQS samples was recorded at 735 nm (*A*_1_), and VC was used as a positive control. Anhydrous ethanol instead of the PQS and BPQS sample was recorded as *A*_0_, while anhydrous ethanol instead of the ABTS working solution was recorded as *A*_2_. The ABTS^+^ RSA was calculated by the following equation:

ABTS^+^ RSA (%) = [1 − (*A*_1_ − *A*_2_)/*A*_0_] × 100

#### 3.6.3. Total Reducing Power

The total reducing power of PQS and BPQS was assessed using the method described in [60,61]. Briefly, 250 μL of the PQS and BPQS sample solution at various concentrations (0.2–1.2 mg/mL) was added with 250 μL of 2 M phosphate buffer (pH = 6) and 250 μL of 1% potassium ferricyanide, and kept at a 50 °C water bath for 20 min. After cooling to room temperature, 250 μL of 10% trichloroacetic solution was added, and then the mixture was centrifuged at 3500 rpm for 10 min. By blending the supernatant (100 μL) with 100 μL distilled water and 20 μL of 0.1% ferric chloride solution, the absorbance of the mixture solution was recorded at 700 nm to quantify the total reducing power.

### 3.7. Determination of In Vitro Cell Cytotoxicity

#### 3.7.1. Cell Viability Assay

HepG2 cells were cultured in a 10% fetal bovine serum (FBS) and 1% penicillin/streptomycin containing Dulbecco’s modified eagle medium at 37 °C in an atmosphere with 5% CO_2_. The cell viability was determined using the CCK-8 kit. The HepG2 cell suspension (100 μL; 5 × 10^3^ cells/well) was seeded into a 96-well plate and cultured in medium overnight. After rinsing twice with PBS solution, 100 μL of PQS and BPQS sample solutions at various concentrations (0, 50, 100, 150, 200, 250, and 300 μg/mL) were added and incubated for 12 h, 24 h, and 48 h, followed by incubation with 10 μL of cell counting kit-8 (CCK-8) at 37 °C for 1.5 h. The absorbance of the PQS and BPQS samples was recorded at 450 nm (*A*_1_). Cell-free and drug-free medium was recorded as *A*_0_, while drug-free medium with HepG2 cells was recorded as *A*_2_. The cell viability was calculated by the following equation:

Cell viability (%) = (*A*_1_ − *A*_0_)/(*A*_2_ − *A*_0_) × 100

#### 3.7.2. Cell Growth State Observation

HepG2 cells (70–80% fusion rate; 25T flask) were resuspended and seeded into a 12-well plate. After 12 h of incubation, the cell-containing well was rinsed twice with PBS solution and then filled with 1 mL of PQS and BPQS sample solution at various concentrations (50–300 μg/mL). The culture medium (1 mL) was used in the control group. After 48 h of incubation, the cell growth state of HepG2 cells was observed under an inverted microscope for recording.

#### 3.7.3. Cell Apoptosis Assay

Cell apoptosis rates of PQS and BPQS against HepG2 cells were determined by flow cytometry using Annexin V-FITC/PI dual staining [28]. HepG2 cells were incubated with PQS and BPQS sample solutions at various concentrations (0–200 μg/mL) for 48 h. Then, HepG2 cells were rinsed with PBS buffer and incubated with EDTA-free 0.25% trypsin at room temperature for 2 min to digest the cells. The digestion reaction was terminated by adding the collected old medium. After centrifugation at 1000 rpm for 5 min, the residue was resuspended in pre-cooled PBS buffer at 4 °C. The cell suspension (5 × 10^4^–1 × 10^5^ cells) was centrifuged at 1000 rpm, and 4 °C for 5 min, and the residue was combined with 195 μL binding buffer. Cells were stained with 5 μL of Annexin V-FITC and 10 μL of PI, allowed to stand in the dark at room temperature for 10–20 min, and then immediately transferred to an ice bath in the dark. Cell apoptosis was evaluated using a CytoFLEX flow cytometry system (Beckman Coulter, Brea, CA, USA) within 1 h.

#### 3.7.4. Western Blot Analysis

The expression levels of apoptosis-related proteins caspase 3 and caspase 9 in HepG2 cells were determined by the Western blot analysis. Briefly, HepG2 cells were seeded into a 6-well plate and cultured overnight until they reached 70–80% fusion rate. Then, cells were treated with PQS and BPQS at various concentrations (0–200 μg/mL) for 48 h, followed by rinsing with PBS. Subsequently, an aliquot of 150 μL lysis buffer (RIPA:1 mM PMSF = 100:1) was added to lyse the cells on ice for 20–30 min. The lysed proteins were centrifuged at 12,000 rpm for 5 min, and the supernatant was collected as the protein extract. The protein concentration was determined using the bicinchoninic acid (BCA) kit procedure. The 96-well plate was shaken evenly and incubated at 37 °C for 30 min, after which the absorbance was measured at 562 nm. The standard curve for determining protein content was y = 0.2725x − 0.0007 (R^2^ = 0.9989).

The protein extract (20 μL) was loaded into each well of a 10% SDS-PAGE separation gel. Electrophoresis was carried out on the Mini-PROTEAN Tetra system (Bio-Rad, Hercules, CA, USA) at 70 V for 30 min, then the voltage was adjusted to 120 V until the sample reached the bottom. The protein strips were transferred to PVDF membrane in 1× Transfer Buffer (2.9 g glycine, 5.8 g Tris, 0.37 g SDS, and 200 mL methanol diluted to 1 L with ultrapure water) with a constant current of 200 mA for 120 min. The transferred PVDF membrane was blocked with blocking solution (5 g skimmed milk powder dissolved in 100 mL TBST buffer) and incubated for 60 min on a shaker at room temperature. The blocked membranes were incubated with the primary antibody solution overnight at 4 °C. After rinsing them in TBST solution three times for 5 min each time, the membranes were incubated with the secondary antibody working solution for 1 h on a shaker at room temperature and washed with TBST buffer three times for 10 min each time. Finally, the protein bands were visualized using an ultrasensitive ECL substrate for 60 s, and then photographed and analyzed with ImageJ 1.54g software (National Institutes of Health, New York, NY, USA). The expression level of the target protein was calculated by normalizing to the internal reference protein GAPDH.

### 3.8. Statistical Analysis

Unless otherwise specified, all data are from independent and repeated experiments (*n* ≥ 3) and are presented as mean ± standard deviation. Data analysis was performed using SPSS 21.0 software (SPSS Inc., Chicago, IL, USA) for one-way ANOVA. Differences were considered statistically significant at *p* < 0.05.

## 4. Conclusions

This study systematically investigated the blackening process of *Panax quinquefolius* L. (PQ) by regulating parameters, including temperature, relative humidity (RH), and treatment time. During blackening, the composition and content of ginsenosides changed dramatically. The potential transformation pathways mainly involved the conversion of protopanaxadiol (PPD)-type ginsenoside Rb1 into Rk1 and Rg5, as well as protopanaxatriol (PPT)-type ginsenoside Re into (S)-Rh1, as determined by LC-IT-TOF-MS/MS analysis. Furthermore, results indicated that compared with PQS, BPQS exhibited better antioxidant activities and stronger cytotoxicity toward HepG2 cells. Considering the transformation of ginsenosides during blackening, the better apoptosis-inducing effect of BPQS may be attributed to the increased content of less-polar ginsenosides such as Rk1 and Rg5, which can promote the expression of apoptosis-related proteins (caspase 3 and caspase 9). Therefore, BPQS holds promise as a functional ingredient for dietary supplements and chemoprevention.

## Figures and Tables

**Table 1:** Total saponin content in blackened *Panax quinquefolius* L. treated at different temperatures (70–90 °C) and relative humidities (RHs, 70–85%) for 0–14 days.

Total Saponin Content (%)		Treatment Time (d)							
Temperature	RH	0	2	4	6	8	10	12	14
70 °C	70%	2.72 ± 0.03 ^aA^	1.24 ± 0.05 ^cF^	1.74 ± 0.08 ^bE^	1.65 ± 0.12 ^bC^	1.21 ± 0.11 ^cE^	1.18 ± 0.04 ^cE^	1.19 ± 0.10 ^cD^	1.10 ± 0.04 ^cH^
	75%	2.72 ± 0.03 ^aA^	1.40 ± 0.10 ^bE^	1.21 ± 0.05 ^bcE^	1.06 ± 0.02 ^cC^	1.19 ± 0.17 ^bcE^	1.12 ± 0.13 ^cE^	1.10 ± 0.04 ^cD^	1.38 ± 0.10 ^bGH^
	80%	2.72 ± 0.03 ^aA^	1.90 ± 0.10 ^bC^	1.21 ± 0.07 ^cE^	1.23 ± 0.12 ^cC^	1.23 ± 0.11 ^cE^	1.14 ± 0.03 ^cE^	1.16 ± 0.13 ^cD^	1.31 ± 0.07 ^cGH^
	85%	2.72 ± 0.03 ^aA^	1.64 ± 0.09 ^cdD^	1.80 ± 0.05 ^bcDE^	1.88 ± 0.30 ^bC^	1.37 ± 0.10 ^fE^	1.48 ± 0.07 ^defE^	1.44 ± 0.08 ^efD^	1.56 ± 0.07 ^deG^
80 °C	70%	2.72 ± 0.03 ^dA^	2.60 ± 0.06 ^dA^	3.04 ± 0.24 ^dBC^	5.21 ± 0.85 ^abA^	4.42 ± 0.16 ^bcB^	4.28 ± 0.28 ^bcAB^	5.73 ± 0.15 ^aA^	3.50 ± 0.09 ^cdDE^
	75%	2.72 ± 0.03 ^bcA^	2.63 ± 0.29 ^bcB^	3.67 ± 0.50 ^aB^	3.30 ± 0.06 ^abB^	3.48 ± 0.29 ^aCD^	3.70 ± 0.14 ^aBCD^	3.22 ± 0.24 ^abC^	2.45 ± 0.18 ^cF^
	80%	2.72 ± 0.03 ^cA^	2.67 ± 0.06 ^cdB^	2.63 ± 0.17 ^cdCD^	2.97 ± 0.09 ^cB^	2.94 ± 0.05 ^cD^	4.14 ± 0.20 ^aABC^	3.54 ± 0.35 ^bBC^	3.96 ± 0.08 ^abCD^
	85%	2.72 ± 0.03 ^deA^	3.20 ± 0.57 ^cdA^	3.71 ± 0.22 ^bcB^	5.29 ± 0.07 ^aA^	5.29 ± 0.32 ^aA^	4.35 ± 0.02 ^bAB^	4.09 ± 0.17 ^bB^	2.39 ± 0.18 ^eF^
90 °C	70%	2.72 ± 0.03 ^eA^	3.67 ± 0.24 ^dA^	4.74 ± 0.44 ^abA^	5.09 ± 0.43 ^aA^	4.01 ± 0.26 ^bcdBC^	3.50 ± 0.40 ^dCD^	3.79 ± 0.07 ^cdBC^	4.53 ± 0.26 ^abcB^
	75%	2.72 ± 0.03 ^dA^	4.42 ± 0.20 ^aB^	4.88 ± 0.24 ^bA^	4.57 ± 0.07 ^abA^	4.01 ± 0.29 ^bcBC^	3.49 ± 0.28 ^cCD^	3.59 ± 0.54 ^cBC^	5.08 ± 0.25 ^aA^
	80%	2.72 ± 0.03 ^dA^	3.35 ± 0.20 ^bcB^	3.16 ± 0.16 ^cdBC^	3.19 ± 0.17 ^cB^	3.56 ± 0.31 ^bcCD^	3.29 ± 0.03 ^cD^	4.00 ± 0.11 ^abB^	4.25 ± 0.16 ^aBC^
	85%	2.72 ± 0.03 ^cA^	3.96 ± 0.29 ^abA^	4.95 ± 0.44 ^aA^	4.88 ± 0.16 ^aA^	4.62 ± 0.46 ^aB^	4.64 ± 0.80 ^aA^	3.55 ± 0.22 ^bcBC^	3.29 ± 0.35 ^bcE^

Note: Results are presented as the mean ± standard deviation. Different lowercase letters in the same row indicate a significant difference (*p* < 0.05); different uppercase letters in the same column indicate a significant difference (*p* < 0.05).

**Table 2:** Identification of ginsenosides in PQS and BPQS.

Peak	Rt (min)	Ginsenosides	Formula	*m*/*z* [M−H]^−^	MS/MS Fragments	Samples
1	34.053	Rg1	C_42_H_72_O_14_	799.4854	637.4330 [M−H−Glc]^−^ 475.3687 [M−H−2Glc]^−^	PQS
2	34.198	Re	C_48_H_82_O_18_	945.5356	799.4837 [M−H−Rha]^−^ 783.4887 [M−H−Glc]^−^ 637.4312 [M−H−Glc−Rha]^−^ 475.3818 [M−H−2Glc−Rha]^−^	PQS, BPQS
3	46.047	Rb1	C_54_H_92_O_23_	1107.5927	945.5381 [M−H−Glc]^−^ 783.4885 [M−H−2Glc]^−^ 621.4341 [M−H−3Glc]^−^ 459.3843 [M−H−4Glc]^−^	PQS, BPQS
4	47.653	Rg2	C_42_H_72_O_13_	783.4890	637.4307 [M−H−Glc]^−^ 475.3800 [M−H−2Glc]^−^	BPQS
5	48.163	(S)-Rh1	C_36_H_62_O_9_	637.4348	475.3813 [M−H−Glc]^−^	BPQS
6	52.245	Rd	C_48_H_82_O_18_	945.5386	783.4855 [M−H−Glc]^−^ 621.4362 [M−H−2Glc]^−^ 459.3874 [M−H−3Glc]^−^	PQS, BPQS
7	65.165	F2	C_42_H_72_O_13_	783.4869	621.4370 [M−H−3Glc]^−^	PQS, BPQS
8	69.057	Rg3	C_42_H_72_O_13_	783.4879	621.4366 [M−H−Glc]^−^ 459.3833 [M−H−2Glc]^−^	BPQS
9	77.037	Rk1	C_42_H_70_O_12_	783.4776	603.4278 [M−H−Glc]^−^	BPQS
10	78.055	Rg5	C_42_H_70_O_12_	765.4775	603.4242 [M−H−Glc]^−^	BPQS
11	79.990	(S)-Rh2	C_36_H_62_O_8_	621.4371	459.3829 [M−H−Glc]^−^	BPQS

## Data Availability

The original contributions presented in the study are included in the article/Supplementary Materials.

## Supplementary Materials

The following supporting information can be downloaded at: https://www.mdpi.com/article/10.3390/molecules31071173/s1, Table S1. The chemical compositions of *Panax quinquefolius* L.; Table S2. Polysaccharide content changes in *Panax quinquefolius* L. under different temperature (70–90 °C) and relative humidity (RH, 70–85%) treatment conditions for 0–14 days; Table S3. Water content changes in *Panax quinquefolius* L. under different temperature (70–90 °C) and relative humidity (RH, 70–85%) treatment conditions for 0–14 days; Table S4. Ginsenosides contents in PQS and BPQS; Figure S1. MS2 analysis of ginsenoside extracts with Protopanaxadiol (PPD)-type ginsenosides Rb1 (A) and Rd (B), and Protopanaxtriol (PPT)-type ginsenosides Re (C) and Rg1 (D) as examples; Figure S2. Cell migration of different concentrations (0–300 μg/mL) of PQS (A) and BPQS (B) against HepG2 cells after incubation for 48 h; Table S5. Cell migration rate of different concentrations (0–300 μg/mL) of PQS (A) and BPQS (B) against HepG2 cells after incubation for 48 h.
