# Supplementary material for: Blackened Panax quinquefolius L. Saponins and Their Cytotoxic Effect on HepG2 Cells
Source: Molecules. 2026 Apr 1;31(7):1173. doi: 10.3390/molecules31071173 (PMC13075200; doi:10.3390/molecules31071173)
Supplement: Supplementary file 1 [file molecules-31-01173-s001.zip › molecules-4203398-supplementary.pdf]

Table S1 The chemical compositions of *Panax quinquefolius* L.

Components	Contents (%)
Fat	1.38±0.26
Ash	2.37±0.01
Polysaccharide	6.11±0.86
Reducing sugar	6.90±0.57
Total sugar	36.67±0.92
Protein	9.73±0.08
Total saponin	2.72±0.03

Table S2 Polysaccharide content changes in *Panax quinquefolius* L. under different temperature (70–90 °C) and relative humidity (RH, 70%–85%) treatment conditions for 0–14 days.

Polysaccharide content (%)		Treatment time (d)							
Temperature	RH	0	2	4	6	8	10	12	14
70 °C	70%	6.11±0.86^{deA}	7.47±1.54^{cdDE}	5.27±0.36^{eE}	9.12±0.11^{bcCD}	9.18±0.51^{bcDE}	10.12±0.61^{bB}	12.83±0.31^{aA}	14.32±0.54^{aA}
	75%	6.11±0.86^{A}	7.18±0.64^{dcDE}	10.38±0.21^{abBC}	9.09±0.63^{bCD}	9.36±0.35^{bDE}	10.30±0.36^{abB}	10.98±0.30^{aBC}	9.84±0.86^{abCD}
	80%	6.11±0.86^{cA}	4.47±0.10^{dG}	8.32±0.41^{bCD}	7.98±0.25^{bcDE}	10.46±0.66^{aCD}	10.52±0.66^{aB}	11.82±0.54^{aAB}	11.01±0.99^{aBC}
	85%	6.11±0.86^{cA}	6.56±0.68^{cEF}	4.79±0.50^{dE}	4.97±0.18^{dF}	6.89±0.16^{cF}	9.80±0.11^{cBC}	10.66±0.12^{aBCD}	11.43±0.20^{aBC}
80 °C	70%	6.11±0.86^{eA}	8.73±1.01^{dCD}	10.95±0.51^{bcAB}	10.83±0.43^{bcB}	11.57±0.31^{bcBC}	14.34±0.14^{aA}	13.06±0.51^{bA}	11.42±1.24^{bcBC}
	75%	6.11±0.86^{eA}	7.23±0.72^{cdDE}	7.36±0.44^{bcdD}	9.13±0.40^{bCD}	8.97±0.31^{bcE}	11.26±0.83^{aB}	12.70±0.87^{aA}	11.50±0.99^{aBC}
	80%	6.11±0.86^{bA}	5.21±0.37^{bFG}	7.49±1.67^{bD}	7.54±0.54^{bE}	13.14±0.73^{aA}	13.20±0.87^{aA}	11.15±0.79^{aB}	12.83±0.01^{aAB}
	85%	6.11±0.86^{eA}	10.73±0.56^{cdAB}	11.83±0.24^{abcAB}	12.65±0.76^{aA}	12.39±0.27^{abAB}	9.83±0.11^{dBC}	10.91±0.72^{bcdBC}	11.44±0.81^{bcdBC}
90 °C	70%	6.11±0.86^{cA}	11.77±0.56^{aA}	8.99±0.75^{bCD}	8.84±0.56^{bDE}	6.42±0.17^{cF}	7.91±0.44^{bD}	5.94±0.28^{cF}	5.25±0.19^{cE}
	75%	6.11±0.86^{dA}	8.95±0.23^{abCD}	10.29±0.17^{aBC}	10.42±0.36^{aBC}	8.90±0.79^{abcE}	7.28±1.24^{cdD}	8.26±0.51^{bcE}	6.45±0.38^{dE}
	80%	6.11±0.86^{cA}	9.87±0.58^{abBC}	10.36±0.92^{eBC}	10.73±0.86^{aB}	10.13±0.06^{abDE}	8.17±0.51^{bCD}	9.58±1.07^{abCDE}	8.84±0.85^{abD}
	85%	6.11±0.86^{eA}	9.29±0.33^{bcBC}	12.61±0.39^{aA}	10.61±0.94^{bBC}	9.33±0.96^{bcDE}	7.96±0.26^{dD}	9.19±0.30^{bcDE}	8.32±0.52^{bcD}

Note: Results are presented as the mean ± standard deviation. Different lowercase letters in the same row indicate a significant difference ($p < 0.05$); Different uppercase letters in the same column indicate a significant difference ($p < 0.05$).

Table S3 Water content changes in *Panax quinquefolius* L. under different temperature (70–90 °C) and relative humidity (RH, 70%–85%) treatment conditions for 0–14 days.

Water content (%)		Treatment time (d)							
Temperature	RH	0	2	4	6	8	10	12	14
70 °C	70%	67.81±0.34^{aA}	57.17±0.35^{bBC}	40.07±0.35^{cB}	40.30±0.79^{cC}	19.55±0.44^{dG}	17.34±0.50^{eE}	18.07±0.75^{eE}	20.46±0.18^{dCD}
	75%	66.40±0.33^{aAB}	63.96±0.92^{bA}	47.41±0.25^{cA}	34.13±0.25^{dE}	24.58±0.20^{fE}	20.97±0.24^{gD}	20.99±0.52^{gD}	31.67±1.24^{eA}
	80%	68.94±0.10^{aA}	59.21±1.65^{bB}	48.23±1.58^{cA}	48.46±0.79^{dA}	33.08±1.37^{eC}	21.55±0.33^{fD}	21.27±0.18^{fD}	21.21±0.24^{fC}
	85%	63.81±0.13^{aB}	47.01±1.70^{bE}	46.95±0.54^{bA}	45.63±0.31^{bB}	42.77±0.67^{cA}	38.06±0.57^{dA}	43.43±0.51^{cA}	25.35±0.36^{eB}
80 °C	70%	54.06±0.71^{aE}	41.81±1.00^{bF}	20.93±0.47^{cE}	19.18±0.52^{cH}	12.56±0.27^{deI}	14.97±0.44^{dF}	11.05±0.13^{eG}	12.11±0.09^{deH}
	75%	60.12±1.91^{aC}	40.28±1.58^{bF}	36.30±0.43^{cC}	32.19±0.73^{dE}	25.28±0.21^{fDE}	24.27±0.75^{fC}	28.96±0.03^{eC}	26.58±0.74^{efB}
	80%	56.42±0.59^{aDE}	53.77±0.78^{bD}	41.64±0.31^{cB}	32.77±0.02^{dE}	26.54±0.68^{eD}	30.10±0.38^{dB}	18.50±0.81^{fE}	18.43±0.12^{fEF}
	85%	58.35±0.24^{aCD}	54.82±0.80^{bCD}	48.53±0.84^{cA}	39.81±0.88^{cD}	41.11±0.74^{dB}	36.88±0.64^{eA}	30.31±0.33^{fB}	19.59±0.16^{gDE}
90 °C	70%	56.54±0.63^{aDE}	32.60±0.09^{bH}	31.07±0.60^{cD}	15.50±0.38^{dI}	9.65±0.25^{eJ}	8.69±0.35^{efH}	7.81±0.60^{fH}	9.64±0.51^{eI}
	75%	58.73±2.69^{aCD}	36.36±0.48^{bG}	31.77±0.24^{cD}	18.68±0.57^{dH}	14.69±0.31^{eH}	12.97±0.03^{efG}	12.12±0.36^{fG}	10.52±0.93^{gI}
	80%	58.39±1.24^{aCD}	45.56±0.48^{bE}	41.46±1.02^{cB}	26.26±0.34^{dF}	20.76±0.27^{eFG}	13.65±0.70^{gG}	13.52±0.05^{gF}	18.04±0.38^{fF}
	85%	60.70±1.78^{aC}	46.05±0.54^{bE}	35.49±0.01^{cC}	22.45±0.45^{dG}	21.19±0.35^{eF}	16.49±0.35^{fE}	20.77±0.09^{eD}	15.38±0.27^{gG}

Note: Results are presented as the mean ± standard deviation. Different lowercase letters in the same row indicate a significant difference ($p < 0.05$); Different uppercase letters in the same column indicate a significant difference ($p < 0.05$).

Table S4 Ginsenosides contents in PQS and BPQS.

Ginsenosides	PQS (mg/g)	BPQS (mg/g)
Rg1	0.68±0.01	ND
Re	11.18±0.01	1.25±0.01
Rb1	10.37±0.62	8.40±0.03
Rg2	ND	1.70±0.01
(S)-Rh1	ND	0.17±0.02
Rd	3.20±0.14	1.75±0.37
F2	0.51±0.34	7.92±0.08
Rg3	ND	1.77±0.17
Rk1	ND	8.89±0.15
Rg5	ND	17.69±0.75
(S)-Rh2	ND	0.27±0.02
Total	25.91±0.12	50.52±1.21

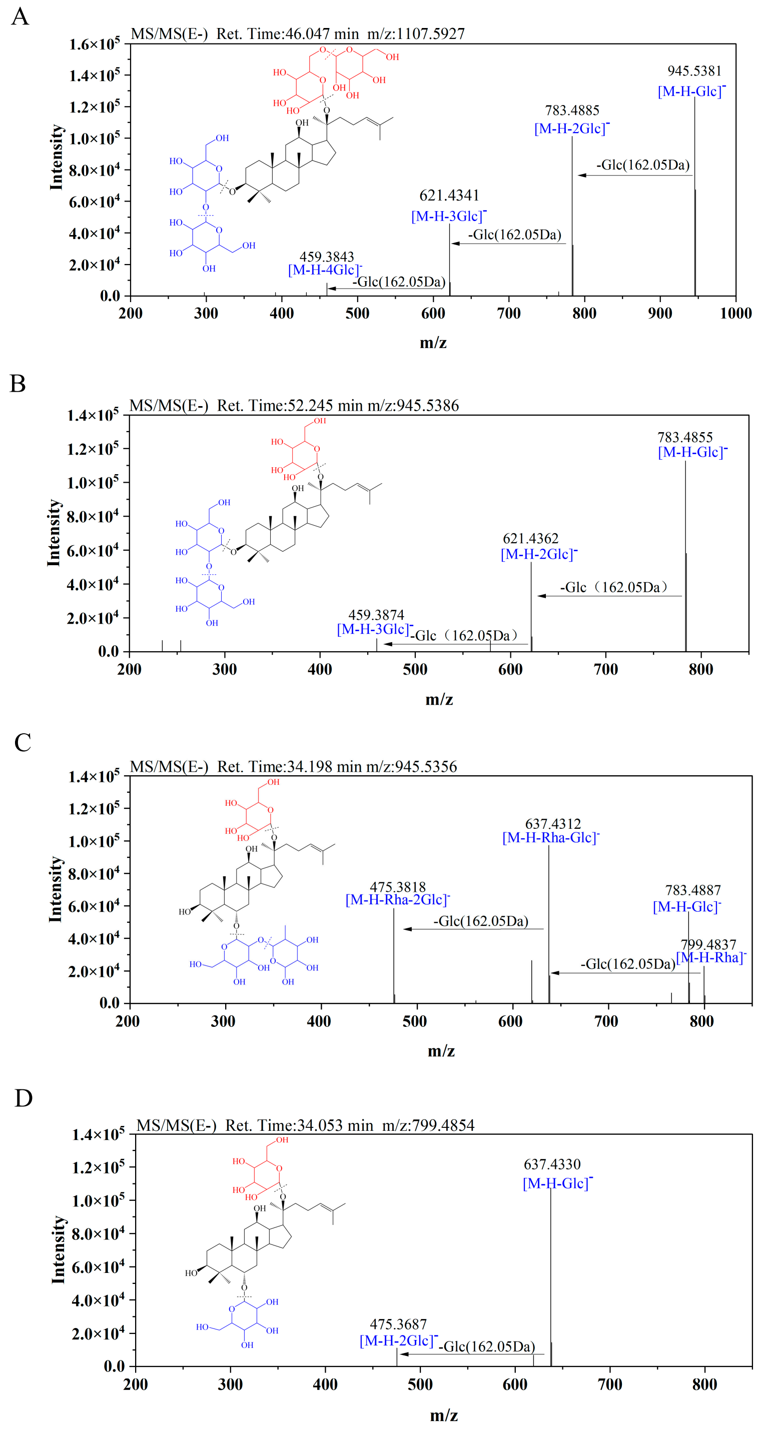

Figure S1. MS^2 analysis of ginsenoside extracts with Propanaxanediol (PPD)-type ginsenosides Rb1 (A) and Rd (B), and Propanaxantriol (PPT)-type ginsenosides Re (C) and Rg1 (D) as examples.

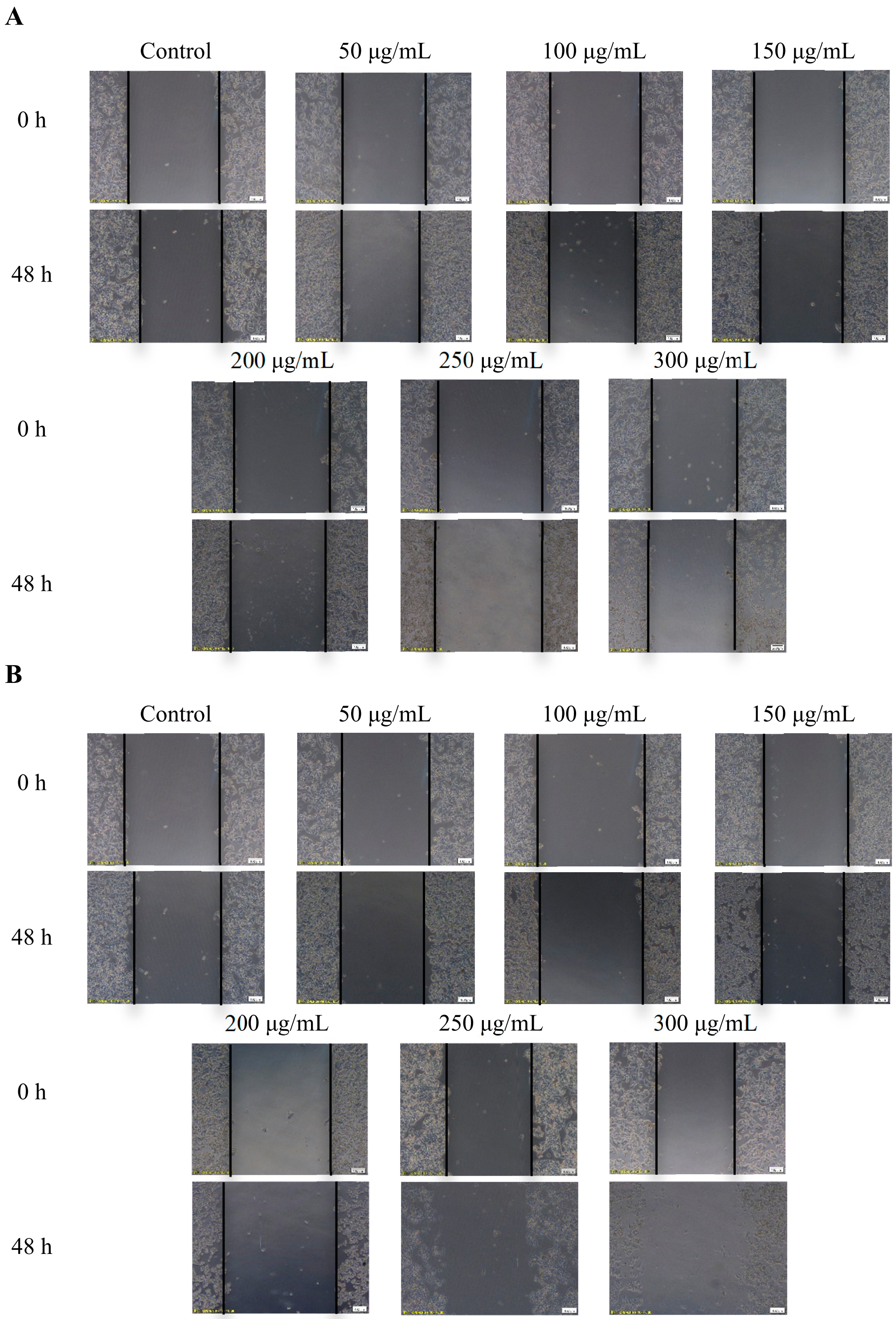

Figure S2. Cell migration of different concentrations (0–300 μg/mL) of PQS (A) and BPQS (B) against HepG2 cells after incubation for 48 h.

Table S5 Cell migration rate of different concentrations (0–300 μg/mL) of PQS (A) and BPQS (B) against HepG2 cells after incubation for 48 h.

Cell migration rate (%)	PQS	BPQS
Control	$9.79±1.02^{aA}$	$9.19±0.36^{aA}$
50 μg/mL	$4.31±0.71^{bA}$	$3.86±0.30^{bA}$
100 μg/mL	$1.58±0.04^{cA}$	$0.94±0.15^{cB}$
150 μg/mL	$1.60±0.53^{cA}$	$-0.63±0.93^{cB}$
200 μg/mL	$0.38±0.25^{cA}$	$-12.53±0.72^{dB}$
250 μg/mL	$-1.89±0.73^{d}$	——
300 μg/mL	$-2.62±0.30^{d}$	——

Note: Results are presented as the mean ± standard deviation. Different uppercase letters in the same row indicate a significant difference ($p < 0.05$); Different lowercase letters in the same column indicate a significant difference ($p < 0.05$).
